# The eco-affective health assessment protocol: a standardized multimodal instrument battery for measuring affective, cognitive, and functional responses to environmental change

**DOI:** 10.3389/fcogn.2026.1881278

**Published:** 2026-08-12

**Authors:** Lucas Murrins Marques

**Affiliations:** 1 Mental Health Department, Santa Casa de Sao Paulo School of Medical Sciences, São Paulo, Brazil; 2 Eco-Wellbeing & Affective Health Laboratory (EWAH Lab), São Paulo, Brazil

**Keywords:** adaptive decision-making, solastalgia, climate psychology, eco-affective health, eco-anxiety, ecological grief, environmental risk perception, multimodal measurement

## Abstract

The field of eco-affective health has produced a growing body of evidence linking environmental change to human psychological and cognitive functioning, but it has done so without a shared methodological foundation. Studies of solastalgia, eco-anxiety, environmental risk perception, and adaptive decision-making use incompatible instruments, inconsistent scoring procedures, and divergent operationalizations of the same constructs, limiting cumulative knowledge, cross-study comparison, and translation into policy-relevant surveillance. This Methods paper introduces the Eco-Affective Health Assessment Protocol (EAHAP): a standardized, multimodal, and modular instrument battery designed to measure affective, cognitive, and functional responses to environmental change in a coherent and replicable framework. The EAHAP organizes existing instruments, the large majority independently validated, with one adapted ecological grief measure explicitly flagged as a candidate instrument pending independent psychometric validation, into three interdependent domains. The eco-affective domain covers solastalgia, eco-anxiety, ecological grief, affective numbing, and place attachment. The cognitive domain covers environmental risk perception, attentional processing, environmental appraisal, and adaptive decision-making under ecological stress. The functional domain covers psychological distress, anxiety and depressive symptoms, sleep disruption, and daily functioning limitation. Each domain is theoretically positioned within the Eco-Affective Health framework as a distinct signal layer, with eco-affective states functioning as early-warning indicators, cognitive mediators explaining variability in response to identical exposures, and functional outcomes providing downstream validation and economic relevance. The paper provides a complete description of materials and instruments, a step-by-step implementation protocol with timing and decision rules, a module selection matrix for different study types, anticipated results with expected psychometric properties, and a troubleshooting guide for the most common pitfalls. Cultural adaptation procedures are specified as a mandatory component of all implementations. The EAHAP is designed to be modular, allowing domain-specific use in fundamental research, and fully integrated for epidemiological surveillance, early warning systems, and intervention evaluation. By providing a shared methodological infrastructure, the EAHAP aims to accelerate the development of eco-affective health as a cumulative, cross-contextual, and policy-relevant scientific field.

## 1 Introduction

The study of how environmental change shapes human psychological experience has reached an important inflection point. Over the past two decades, a substantial and rapidly growing body of research has documented that climate-related exposures, including not only acute events such as floods, wildfires, and heatwaves but also the slower, cumulative deterioration of familiar environments, produce measurable effects on human affect, cognition, and behavior (Clayton, 2020; Pihkala, 2022; Cunsolo and Ellis, 2018). Constructs once confined to the margins of environmental psychology, such as solastalgia, eco-anxiety, and ecological grief, have moved into the mainstream of climate-health discourse (Albrecht, 2007), and there is now broad recognition that the psychological and cognitive dimensions of the human response to environmental change are not peripheral phenomena but central mechanisms through which climate impacts on health, behavior, and social functioning unfold (Brosch, 2021; Weber, 2016; Marques and Franco, 2025).

Yet this conceptual progress has not been matched by full methodological coherence, though it should be acknowledged that meaningful psychometric work has been conducted on several individual constructs within this field, including the development and validation of instruments such as the Hogg Eco-Anxiety Scale Hogg et al., (2021). The field of eco-affective health as a whole, however, remains characterized by a proliferation of instruments that measure related constructs through heterogeneous operationalizations, scoring conventions, and item formats, a pattern that complicates cross-study comparison even where individual instruments are well validated. Solastalgia alone has been measured using at least six distinct instruments across the published literature, with no consensus on item content, response format, or scoring approach (Galway et al., 2019). Eco-anxiety, while increasingly distinguished from climate anxiety in some conceptual frameworks, for example (Hogg et al. 2021), who define eco-anxiety as a broader multidimensional response to the environmental crisis rather than a synonym for climate-specific worry, continues to be operationalized heterogeneously across the literature, appearing variously as a subscale of general anxiety, a trait-level disposition, and a state-level response, with the Climate Anxiety Scale (Clayton and Karazsia, 2020), the Hogg Eco-Anxiety Scale (Hogg et al., 2021), and numerous *ad hoc* measures circulating simultaneously without systematic comparison. Environmental risk perception is measured in ways that conflate its affective and cognitive components, despite compelling evidence that these components predict different downstream outcomes through different mechanisms (Slovic, 2000; van der Linden, 2015). Adaptive decision-making under ecological stress, a theoretically central mechanism linking exposure to behavioral response, is rarely measured directly at all.

This fragmentation has concrete consequences. It prevents meta-analytic synthesis across studies. It makes cross-cultural and cross-contextual comparison unreliable. It produces conflicting findings about the relationships between constructs that may reflect measurement inconsistency rather than genuine empirical variation. And it impedes the translation of eco-affective research into policy-relevant surveillance systems, because decision-makers cannot act on a body of evidence organized around incompatible instruments. What the field requires is not another instrument for any single construct, but a shared methodological infrastructure: a protocol that organizes a battery of predominantly validated instruments, together with one adapted candidate measure pending independent validation (see Table 2), into a coherent, theoretically grounded, and practically implementable assessment framework.

**Figure 1:**
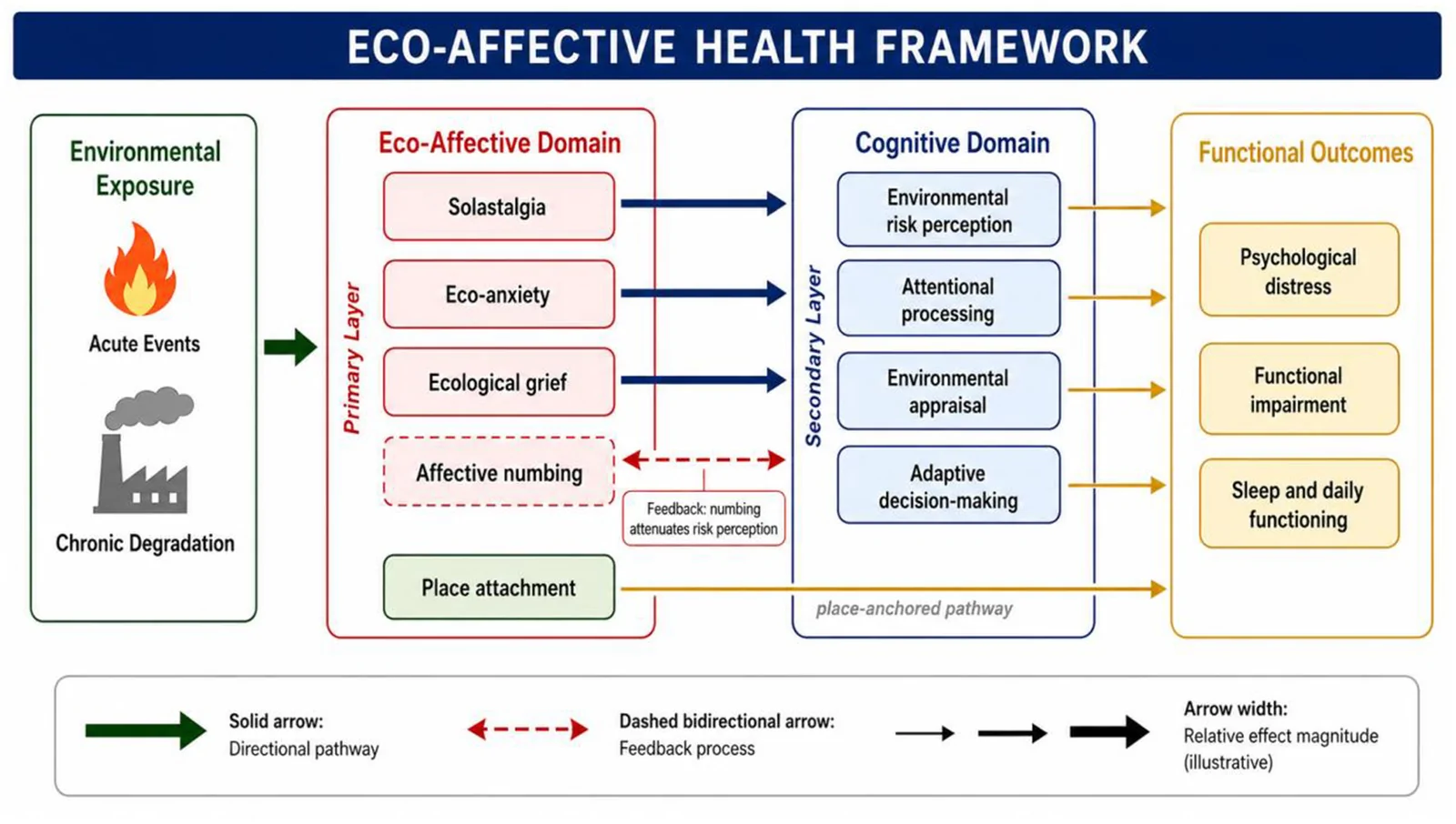
Expected pathway relationships among EAHAP domains and their constituent constructs. Arrows indicate theoretically predicted directional relationships; dashed arrows indicate feedback processes. Arrow widths are illustrative of expected relative effect magnitudes based on existing literature. Eco-affective states are positioned as the primary mediating layer between environmental exposure and functional outcomes; cognitive mediators are positioned as moderators and secondary mediators of this relationship. The bidirectional arrow between affective numbing and cognitive risk perception reflects the expected feedback whereby progressive numbing attenuates risk perception, thereby reducing the motivational force of cognitive appraisal. The comparatively thick arrow drawn from place attachment reflects its expected effect specifically on the proximal, place-anchored form of eco-affective distress that solastalgia represents (Marques, 2026c), and is not intended to suggest that place attachment is the dominant pathway for the full range of eco-affective experience: ecological grief and eco-anxiety are expected to arise, and in some cases to arise most strongly, in relation to ecosystems and places to which a person is not personally or residentially attached, through pathways that bypass place attachment entirely.

This paper introduces the Eco-Affective Health Assessment Protocol (EAHAP) as that infrastructure. The EAHAP draws on the Eco-Affective Health framework (Marques, 2026a), which positions affect not as a downstream reaction to environmental conditions but as a primary regulatory mechanism through which humans perceive, interpret, and respond to the living world. Within this framework, eco-affective states, cognitive mediators, and functional outcomes are not independent variables to be measured in isolation but interdependent signal layers that together describe the human response to environmental change across its full range: from early affective sensing through cognitive processing and appraisal to behavioral regulation and functional impact. This affect-as-regulatory-mechanism premise is not proposed here for the first time: it is the organizing claim of a peer-reviewed theoretical account of Eco-Affective Health (Marques, 2026a) that derives, from the same premise, testable hypotheses regarding the ordering of affective and cognitive signal layers. The ordering adopted in the EAHAP architecture, in which eco-affective states are positioned as proximal to environmental exposure and cognitive mediators as positioned downstream, is not intended to displace appraisal-based models such as Lazarus and Folkman (1984); rather, appraisal processes are treated as continuously interacting with, and in part shaped by, prior affective states, consistent with evidence that affective responses to environmental information can precede and bias subsequent cognitive appraisal (Brosch, 2021; Weber, 2016). This is also why the EAHAP architecture already incorporates a feedback pathway, illustrated in Figure 1, whereby affective numbing attenuates cognitive risk perception, allowing for dynamic, bidirectional influence between the eco-affective and cognitive domains rather than a strictly unidirectional cascade. The EAHAP operationalizes each of these layers with validated instruments selected for their psychometric properties, brevity, cultural adaptability, and compatibility with both fundamental research and applied surveillance contexts.

The EAHAP has three primary applications. First, it provides a standardized instrument battery for fundamental research on eco-affective and cognitive responses to environmental change, enabling cumulative knowledge and cross-study comparison. Second, it constitutes the human signal layer of the Eco-Affective Early Warning System (EAEWS), a multimodal surveillance framework that links spatially explicit environmental exposure indices to human eco-affective and cognitive signals at policy-relevant geographic scales (Marques, 2026b, a forthcoming book under contract and currently in preparation, cited here only for the description of the EAEWS surveillance architecture and not as theoretical support for the EAHAP's own domain structure, which rests instead on the peer-reviewed account in Marques, 2026a). Third, it provides a replicable assessment tool for evaluating interventions in climate adaptation, ecological restoration, and community resilience programs. Across all applications, the EAHAP is designed to be modular, allowing domain-specific implementation in resource-limited contexts, and fully integrated when epidemiological completeness is required.

## 2 Materials and equipment

### 2.1 Instrument battery

The EAHAP comprises 19 validated or adapted instruments organized across three domains. Table 1 presents the full taxonomic structure of the constructs measured by the protocol, including operational definitions, distinctions from adjacent constructs, and positioning within the EAHAP signal architecture. Table 2 provides the complete instrument matrix with item counts, estimated administration times, validation references, and cultural adaptation notes.

**Table 1:** Taxonomic structure of Eco-Affective Health Assessment Protocol (EAHAP) constructs, including operational definitions, distinctions from adjacent constructs, and positioning within the signal architecture.

Construct	Operational definition	Distinction from adjacent constructs	Position in EAHAP
Solastalgia	Distress arising from the lived experience of unwanted environmental change in one's home environment; grief tied to place still inhabited	Distinct from nostalgia (loss of a past place) and displacement distress (loss of access to place); specifically relational and present-tense	Eco-affective domain: primary early-warning signal of eroding human-environment bond
Eco-anxiety	Chronic, anticipatory worry about present and future ecological conditions; includes physiological arousal and functional interference	Distinct from generalized anxiety disorder by its environmental object and adaptive function; not inherently pathological	Eco-affective domain: forward-looking distress signal; moderates engagement vs avoidance
Ecological grief	Mourning response to perceived or actual loss of species, landscapes, seasonal rhythms, or ecological relationships	Distinct from clinical depression; episodic rather than persistent; often collectively experienced and culturally mediated	Eco-affective domain: signals depth of ecological attachment; predicts long-term stewardship motivation
Affective numbing	Progressive attenuation of emotional responsiveness to environmental information as a regulatory strategy under chronic threat	Distinct from resilience; reflects regulatory exhaustion rather than adaptive coping; associated with disengagement rather than recovery	Eco-affective domain: late signal of affective overload; indicates risk of stewardship withdrawal
Place attachment	Affective bond between a person and a specific environment, built through repeated experience and social meaning	Distinct from general nature connectedness; place-specific and spatially anchored; functions as a vulnerability amplifier for solastalgia	Eco-affective domain: moderating variable; amplifies solastalgic response when degradation occurs in high-attachment places
Environmental risk perception	Multidimensional subjective assessment of probability, severity, and personal relevance of environmental threats; includes affective and cognitive components	Distinct from objective risk; the affective component (felt danger) and cognitive component (estimated probability) are analytically separable and predict different outcomes	Cognitive domain: primary gateway through which exposure translates into affective and behavioral response
Attentional processing	Selective attention to environmental threat information; includes scanning behavior and avoidant attention as regulatory strategies	Distinct from general attentional capacity; specifically environmental in object; varies with exposure history and affective state	Cognitive domain: mediates salience of environmental conditions; avoidant attention predicts disengagement trajectories
Environmental appraisal	Two-stage evaluative process: primary appraisal of threat magnitude and secondary appraisal of coping capacity and available resources	Distinct from risk perception by its action-oriented focus; secondary appraisal is the key determinant of adaptive vs avoidant behavioral response	Cognitive domain: explains variability in behavioral response to identical exposure and affective conditions
Adaptive decision-making	Capacity to engage in pro-environmental and adaptive behaviors under conditions of environmental stress; includes resistance to fatalism and decision fatigue	Distinct from behavioral intention; specifically captures degradation of decision capacity under chronic stress, not merely motivation	Cognitive domain: downstream cognitive outcome; links appraisal and affect to observable behavioral trajectories
Psychological distress	Transdiagnostic indicator of general mental health burden; sensitive to subclinical changes across multiple symptom domains	Distinct from diagnosis; used as continuous screening measure rather than categorical classification; captures burden before clinical threshold	Functional domain: downstream validation target; confirms that upstream eco-affective signals predict broader mental health burden
Functional impairment	Restriction in daily activities, work capacity, and social functioning attributable to eco-affective and cognitive strain	Distinct from clinical disability; captures everyday functional costs below clinical and occupational thresholds; economically relevant	Functional domain: translates eco-affective burden into observable, measurable, and economically quantifiable consequences

Constructs are organized by domain (Eco-Affective, Cognitive, and Functional) and positioned within the EAHAP signal architecture.

Distinctions from adjacent constructs are provided to guide item selection and prevent conceptual overlap in participant briefings.

All constructs are treated as continuous dimensions rather than categorical states; clinical cut-offs are not applied in non-clinical research or surveillance contexts.

**Table 2:** Complete instrument matrix of the Eco-Affective Health Assessment Protocol (EAHAP), including instruments, item counts, estimated administration times, validation references, and cultural adaptation notes.

Construct	Instrument	Items	Time (min)	Validation reference	Adaptation notes
Eco-affective domain					
Solastalgia	Brief Solastalgia Scale (BSS)	5	3–4	Christensen et al. (2024)	Validated 5-item short form of the Environmental Distress Scale solastalgia subscale, with excellent model fit and cross-sample invariance (Christensen et al., 2024). Independently validated translations already exist in Spanish, adapted for Colombian Indigenous communities (Agudelo-Hernández and Guapacha-Montoya, 2025), and in Brazilian Portuguese (Marques et al., 2026). Adapters working in other languages should locate and evaluate an existing validated translation before undertaking fresh forward-translation, consistent with the general guidance in section 3.5.
Eco-anxiety	Climate anxiety scale	13	5–6	Clayton and Karazsia (2020)	Distinguish from generalized anxiety in briefing; functional impairment subscale critical
Ecological grief	Ecological grief scale (adapted)	6	3	Cunsolo and Ellis (2018); adapted items	Loss object must be culturally grounded; allow open specification of loss referent. Items are adapted from the conceptual account of ecological grief in (Cunsolo and Ellis 2018), which does not itself provide a standardized psychometric instrument. Unlike the Brief Solastalgia Scale, no independently validated instrument yet exists for this construct: this measure remains a candidate adaptation requiring independent validation prior to use as a stand-alone instrument.
Affective numbing	Environmental numbing items (adapted)	5	2	Adapted from emotional numbing literature; see Pihkala (2020)	Reverse-scored items require careful translation; pilot for comprehension
Place attachment	Place attachment scale (short form)	9	4	Scannell and Gifford (2010)	Requires stable place of residence as anchor; adapt for displaced or mobile populations
Cognitive domain					
Environmental risk perception	Environmental risk perception Scale (affective + cognitive + temporal)	12	5	Slovic (2000); van der Linden (2015); adapted	Three subscales must be scored separately; do not collapse into single score
Attentional processing	Environmental attention and avoidance items	8	3	Adapted from attentional bias literature	Self-report proxy; behavioral task optional for laboratory settings
Environmental appraisal	Environmental appraisal items (primary + secondary)	10	4	Adapted from Lazarus and Folkman (1984)	Secondary appraisal items (coping capacity) are the most predictive; do not omit
Adaptive decision-making	Ecological behavior intention and fatalism scale	10	4	Adapted from pro-environmental behavior literature; see Gifford and Chen (2017)	Fatalism items require cultural sensitivity; avoid language implying individual blame
Functional domain					
Psychological distress	Kessler psychological distress scale (K6)	6	2–3	Kessler et al. (2002)	Use as continuous measure; do not apply clinical cut-offs in non-clinical samples
Anxiety symptoms	Generalized anxiety disorder-7 (GAD-7)	7	3	Spitzer et al. (2006)	Use as continuous screening measure; distinguish from eco-anxiety in participant briefing
Depressive symptoms	Patient health questionnaire-9 (PHQ-9)	9	3–4	Kroenke et al. (2001)	Use as continuous screening measure; monitor for floor effects in non-clinical samples
Sleep quality	Pittsburgh sleep quality index (ultra-short, 4-item)	4	2	Buysse et al. (1989); validated short version	Anchor to past 30 days; align with EEI exposure window where applicable
Functional impairment	Daily functioning and productivity limitation items	6	2–3	Adapted from WHO disability assessment schedule	Specify referent period; align with exposure window; avoid clinical disability framing

Item counts refer to the standard validated version of each instrument. Adaptations for ecological momentary assessment (EMA) use ultra-short versions of 4–6 items per construct. Administration times are estimated for self-completion formats; interviewer-administered formats may require additional time. Total estimated time for the full protocol is 50–65 min. Domain-specific modules can be administered in 15–25 min.

Instruments marked as adapted have been modified from original sources with construct definition and item content preserved; full adaptation procedures are described in section 4.5.

### 2.2 Infrastructure requirements

The EAHAP can be administered in paper-and-pencil or digital formats. For digital administration, compatible platforms include REDCap, Qualtrics, and equivalent survey systems capable of routing logic, skip patterns, and geocoding. For studies requiring spatial linkage with environmental exposure data, the platform must support indirect geocoding, in which participants self-identify their census tract, postal code, or equivalent administrative unit, without collecting or storing individual-level address data. The linkage between participant spatial units and the Environmental Exposure Index (EEI) is performed at the aggregate level after individual data are stripped of any identifying geographic information.

For ecological momentary assessment studies, participants require access to a smartphone with notification capability. Study teams require a platform capable of scheduling randomized or fixed-interval notifications, delivering ultra-short item sets, and storing time-stamped responses. For studies in populations with limited smartphone access, validated paper-based EMA alternatives using scheduled diary completion are acceptable.

All implementations require ethics committee approval, informed consent procedures compliant with national research governance frameworks, and a data management plan specifying procedures for separation of identifiable and analytical data, pseudonymization of longitudinal participant identifiers, and secure storage. Studies that collect geocoded data must include explicit consent items covering spatial data use and must specify the level of geographic precision collected.

## 3 Methods

### 3.1 Objectives and validation of the method

The primary objective of the EAHAP is to enable standardized, replicable, and cross-contextually comparable measurement of the affective, cognitive, and functional dimensions of the human response to environmental change. Each instrument included in the protocol satisfies four selection criteria: demonstrated construct validity in peer-reviewed publications, sufficient brevity for field implementation, sensitivity to subclinical variation below clinical thresholds, and established or establishable cross-cultural adaptability. Instruments failing any of these criteria were excluded even when they measured theoretically relevant constructs, on the grounds that methodological rigor cannot be compromised for conceptual coverage. In practice, these criteria were applied sequentially rather than as a simultaneous checklist: construct validity was screened first, against the peer-reviewed psychometric literature for each construct, to generate a shortlist of eligible instruments; brevity and subclinical sensitivity were then used to select among eligible instruments where more than one satisfied the validity criterion, prioritizing the shortest validated version reporting adequate performance below clinical cut-offs; cross-cultural adaptability was assessed last, based on whether an instrument had already been adapted and validated in more than one linguistic or cultural context, or, where this was not yet available, whether its item content was judged conceptually portable across contexts pending the adaptation procedure specified in section 3.5. Instruments for which no version satisfied all four criteria simultaneously, most notably ecological grief, is flagged explicitly in Table 2 as an adapted measure requiring further validation, rather than presented as a fully validated instrument.

The integration architecture of the EAHAP is validated by its correspondence to the theoretical pathway model of the Eco-Affective Health framework. In this model, environmental exposures generate eco-affective states through processes of appraisal and meaning-making; these states are modulated by cognitive mediators including risk perception, attentional processing, and coping appraisal; and the combined effect of affective states and cognitive processing produces functional outcomes that are measurable, economically relevant, and predictive of longer-term health and behavioral trajectories (Marques, 2026a). The three-domain structure of the EAHAP is not an arbitrary classification but a reflection of this theoretical architecture, and the expected empirical relationships between domains, described in section 5, provide the primary basis for concurrent validity assessment across implementation sites.

### 3.2 Step-by-step procedures

The EAHAP implementation is organized into three phases: preparation, data collection, and processing. Table 3 provides a complete step-by-step overview with timing and pause points. The sections below describe each step in detail.

**Table 3:** Step-by-step Eco-Affective Health Assessment Protocol (EAHAP) implementation procedures, including timing and pause points with decision rules.

Phase	Step	Procedure	Timing	Pause points and decision rules
Phase 1: preparation	Step 1: module selection	Determine which EAHAP modules are required for the study objectives. Full protocol for epidemiological or EAEWS-linked studies; domain-specific modules for focused investigations.	Week 1	No pause point. Module selection is guided by Table 4 decision matrix.
	Step 2: cultural and linguistic adaptation	Forward translation by bilingual specialist; back-translation by independent translator; expert review by local environmental psychologist; cognitive interviewing with 5-10 participants from target population.	Weeks 2–4	PAUSE: do not proceed if cognitive interviews reveal semantic discrepancies in more than 20% of items in any module. Return to expert review and re-interview.
	Step 3: Platform configuration	Configure data collection platform. Set up indirect geocoding (census tract or neighborhood, not address). Configure immediate separation of identifiable and analytical data. Test routing logic and skip patterns.	Weeks 4–6	PAUSE: Do not proceed if geocoding produces linkage errors in test runs. Verify spatial unit alignment with EEI data before proceeding.
	Step 4: Pilot study	Administer full protocol to 30-50 participants from target population. Assess mean completion time, module dropout rates, and item-level non-response. Collect qualitative feedback on clarity.	Weeks 6–8	PAUSE: If completion time exceeds 30 minutes, review item wording. If dropout in any module exceeds 15%, revise wording before full-scale collection.
Phase 2: Data collection	Step 5: Standardized module administration	Administer modules in fixed order: (1) eco-affective, (2) cognitive, (3) functional. Include standardized participant briefing distinguishing eco-affective constructs from clinical diagnoses.	Ongoing	No pause point during collection. Monitor response quality in real time using platform analytics.
	Step 6: Longitudinal re-administration (if applicable)	Re-administer at 30, 60, and 90 days post-baseline. Use rotating pseudonymized identifiers for participant tracking. Align re-administration windows with EEI exposure windows.	Days 30, 60, and 90	PAUSE: If attrition between waves exceeds 25%, assess attrition patterns for bias before proceeding to wave 3.
	Step 7: EMA administration (if applicable)	Use ultra-short modules (4-6 items). Maximum 3 notifications per day. Rotate domains across days. Validate EMA scores against full-protocol baseline in subsample of *n* = 50.	Daily, per EMA protocol	PAUSE: If EMA-baseline concordance falls below *r* = 0.60 for any domain, review EMA item selection and notification timing.
Phase 3: Processing	Step 8: Scoring	Score each instrument per original validation specifications. Apply missing data rules: impute with item mean for up to 20% missing per scale; mark as invalid above 20%.	Post-collection	No pause point. Flag invalid records for sensitivity analysis.
	Step 9: Composite scoring	Standardize domain scores (z-scores within sample). Construct composite eco-affective, cognitive, and functional indices. Document weighting decisions.	Post-collection	PAUSE: If internal consistency of any composite falls below alpha = 0.70, review item composition before using composite in primary analyses.
	Step 10: Environmental linkage (if applicable)	Match participant spatial unit to EEI scores for corresponding exposure window (30, 60, or 90 days prior to collection). Verify linkage completeness before dose-response modeling.	Post-collection	PAUSE: If linkage completeness falls below 85%, investigate missingness patterns and assess impact on dose-response analyses.

Timing estimates are approximate and will vary with sample size, platform configuration, and local administrative requirements. Pause points indicate mandatory decision gates at which implementation must stop pending resolution of the specified condition before proceeding. EMA: ecological momentary assessment.

EEI, Environmental Exposure Index.

K6, Kessler Psychological Distress Scale.

It is worth making explicit, beyond Table 3, the level of participant and staff commitment implied by Phase 2, Step 6: re-administration of the EAHAP at Days 30, 60, and 90 corresponds to one full protocol administration per month for 3 consecutive months, in addition to any ongoing EMA sampling. Study teams planning field implementation should budget for this recurring monthly commitment explicitly in participant retention planning and staffing schedules, rather than treating Step 6 as a single follow-up event.

### 3.3 Module selection

The EAHAP is designed as a modular system. Not all studies require the full protocol, and domain-specific implementations are methodologically valid for studies with appropriately scoped research questions. Table 4 provides a module selection matrix specifying recommended configurations for seven common study types. The decision to use domain-specific rather than full protocol administration should be made before data collection begins and documented in the study protocol, as *post-hoc* module selection introduces analytic flexibility that undermines replicability.

**Table 4:** EAHAP module selection matrix for common study types, specifying required, recommended, and optional domain administrations.

Study type	Eco-affective module	Cognitive module	Functional module	Notes
Fundamental research: eco-affective states and environmental exposure	Required (full)	Recommended	Optional	Minimum viable protocol for EEI dose-response studies
Fundamental research: cognitive mechanisms of environmental risk perception	Recommended	Required (full)	Optional	Risk perception and appraisal modules are the analytical core
Fundamental research: behavioral response to environmental change	Recommended	Required (full)	Recommended	Adaptive decision-making module is essential
Epidemiological surveillance: population-level mental health monitoring	Required (full)	Recommended	Required (full)	Full protocol required for economic impact estimation
EAEWS-linked study: dose-response modeling with EEI	Required (full)	Required (full)	Required (full)	Full protocol mandatory for early warning signal detection
Intervention evaluation: climate adaptation or reconnection programs	Required (full)	Recommended	Recommended	Pre-post design; eco-affective domain is primary outcome
Rapid assessment: resource-limited contexts	Required (core: solastalgia + eco-anxiety)	Recommended (risk perception only)	Optional (K6 only)	Minimum: 25 items, approximately 10 minutes

Required modules must be included for the study results to be interpretable within the EAHAP framework. Recommended modules substantially increase analytic power and interpretability and should be included unless resource constraints prohibit. Optional modules add value in specific analytic contexts but do not compromise the primary research question if omitted. EAEWS: Eco-Affective Early Warning System.

K6: Kessler Psychological Distress Scale.

### 3.4 Administration order and participant briefing

The standardized administration order is: (1) eco-affective domain, (2) cognitive domain, (3) functional domain. This order reflects the theoretical pathway structure of the Eco-Affective Health framework, in which affective states precede and shape cognitive processing, and both upstream processes produce functional outcomes. Administering the functional domain before the eco-affective domain risks priming participants with clinical symptom framing, which can inflate responses on eco-affective scales that are designed to capture non-pathological adaptive responses to environmental conditions.

A standardized participant briefing must precede administration of any EAHAP module. The briefing has three mandatory components: (a) a clear statement that the study is measuring psychological responses to environmental conditions, not diagnosing clinical disorders; (b) a brief explanation of the distinction between eco-affective experiences and mental health diagnoses, using accessible language; and (c) information about how to access support if participation evokes distressing memories or feelings. The exact wording of the briefing is not prescribed, to allow cultural adaptation, but all three components must be present and the briefing must be reviewed by at least one member of the local research team with training in environmental psychology or related fields.

### 3.5 Cultural adaptation procedure

The original validation samples for the instruments in Table 2 are concentrated in a small number of national and linguistic contexts: predominantly the United States (the Climate Anxiety Scale, K6, GAD-7, PHQ-9, and Pittsburgh Sleep Quality Index, and Slovic's contribution to the Environmental Risk Perception Scale), Canada (the conceptual basis for the Ecological Grief Scale, the Place Attachment Scale, and the Ecological Behavior Intention and Fatalism Scale), Australia (the Brief Solastalgia Scale), and the United Kingdom and Netherlands (van der Linden's contribution to the Environmental Risk Perception Scale), with the WHO Disability Assessment Schedule underlying the functional impairment items constituting the principal exception as a genuinely multi-country validated instrument. No instrument in the core battery was originally validated in a Latin American, Sub-Saharan African, or South or Southeast Asian sample. This concentration is not unusual for the eco-affective and environmental psychology literature, but it means the psychometric properties anticipated in section 4.1 should be read as expectations anchored in these specific validation contexts rather than as claims of universal measurement invariance; it is the specific reason the cultural adaptation procedure below is mandatory, rather than optional, for any implementation outside these original contexts. Cultural adaptation is a mandatory component of all EAHAP implementations outside the original validation context of each instrument. The adaptation procedure follows a structured sequence that balances fidelity to the original construct definition with responsiveness to local linguistic and cultural context. The sequence comprises five steps: (1) forward translation of all items by a bilingual specialist with expertise in psychology and environmental science; (2) independent back-translation by a second bilingual specialist with no access to the original; (3) expert review by a committee of at least three members including a local environmental psychologist, a community health researcher, and a bilingual language specialist, with attention to semantic equivalence, cultural resonance, and construct relevance; (4) cognitive interviewing with 5–10 members of the target population using think-aloud protocols to identify comprehension difficulties and culturally incongruent items; (5) psychometric pilot with 30-50 participants to assess internal consistency and factor structure of adapted instruments before full-scale implementation. This five-step sequence is designed to satisfy the requirements of the three principal international frameworks governing test adaptation and evaluation: the International Test Commission's ITC Guidelines for Translating and Adapting Tests (International Test Commission, 2017), which structure steps (1) through (4); the Standards for Educational and Psychological Testing (American Educational Research Association, 2014), whose validity and fairness criteria inform the expert review and cognitive interviewing stages; and the EFPA Review Model for the Description and Evaluation of Psychological Tests (European Federation of Psychologists Associations, 2013), which structures the psychometric pilot in step (5) and the reporting format used in Table 2. Where a validated translation of a given instrument already exists in the target language, for example published adaptations of the Climate Anxiety Scale, the GAD-7, or the PHQ-9 across numerous languages, adapters should locate and evaluate that existing translation against local cultural referents before undertaking fresh forward-translation from the original English source, and should document in study materials why an existing validated translation was or was not adopted. The Hogg Eco-Anxiety Scale is a useful illustration of how quickly this landscape can change: independently validated language versions already exist in German (Heinzel et al., 2023), French (Mathé et al., 2025), Italian (Spano et al., 2025), and Persian (Hasani and Jafari, 2026), in addition to the original English version (Hogg et al., 2021). Adapters working in any of these languages should evaluate these existing validated versions before undertaking fresh translation, and, because new validated translations of eco-affective instruments continue to appear, adapters working in any language should check for recently published versions at the time of implementation rather than assuming the absence of a prior translation. Forward-translation from the original English source should be treated as a fallback for constructs and languages lacking a prior validated version, which at the time of writing includes the Ecological Grief Scale (adapted), for which no prior translated or independently validated version exists in any language.

Particular attention is required for constructs whose referents are culturally variable (Table 5). Solastalgia items must specify the relevant environmental context explicitly, as the sources of place-based distress vary markedly across urban, rural, agricultural, coastal, and Indigenous contexts. Place attachment items require a stable reference environment and must be adapted for populations with disrupted place histories, including displaced persons, refugees, and communities experiencing rapid land-use change. Ecological grief items must allow participants to specify the nature of the loss, as the objects of ecological mourning, whether species, seasonal patterns, water bodies, or agricultural landscapes, vary significantly across cultural contexts and must be locally grounded to capture genuine ecological experience rather than abstract concern.

**Table 5:** Common pitfalls in EAHAP implementation, with likely causes, prevention strategies, and troubleshooting measures.

Pitfall	Likely cause	Prevention	Troubleshooting
High dropout in cognitive module	Items perceived as abstract or cognitively demanding by participants unfamiliar with environmental psychology concepts	Simplify item language in adaptation process; include worked example in participant briefing	Review item wording; reduce cognitive demand without altering construct; add contextual anchoring
Systematic missing data on place attachment items	Participants without stable place of residence or with disrupted place history cannot anchor responses	Add open-ended item to identify reference place before scale administration	Create adapted version anchored to most recent stable environment; flag in analysis
Low internal consistency in eco-anxiety scale	Cultural variation in expression of anticipatory worry; items referencing specific climate events may not resonate	Conduct cognitive interviews specifically on anticipatory worry items; review cultural salience	Remove lowest-loading items after pilot; replace with locally resonant items preserving construct definition
Inflation of psychological distress scores in high-EEI areas	Genuine signal of elevated burden rather than measurement artifact	Include socioeconomic covariates in all dose-response models; assess confounding systematically	Conduct stratified analyses; examine whether inflation persists after covariate adjustment
Poor EMA-baseline concordance	State-level EMA items capture momentary affect; trait-level baseline items capture stable dispositions	Distinguish state and trait framings explicitly in EMA item selection; validate in 50-person subsample	Use multilevel modeling that separates within-person and between-person variance
Geocoding linkage failure	Participant-reported spatial units do not match administrative unit boundaries in EEI dataset	Harmonize spatial unit definitions before data collection; provide visual map for participant self-identification	Apply nearest-neighbor spatial matching for unmatched units; document and report linkage rate
Solastalgia and ecological grief items loading on same factor	Constructs share affective valence; items may not be sufficiently distinct	Ensure item wording emphasizes place-specificity for solastalgia and loss-specificity for grief	Use bifactor modeling to separate general eco-affective distress factor from specific construct factors

Pitfalls are organized by implementation phase. Prevention strategies should be applied during protocol design and pilot stages; troubleshooting measures apply when a pitfall is identified after full-scale data collection has begun. EMA: ecological momentary assessment.

EEI, Environmental Exposure Index.

### 3.6 Scoring procedures

Each instrument is scored according to the specifications of its original validated version, with the following protocol-level conventions. All scores are treated as continuous rather than categorical; clinical cut-offs are not applied in non-clinical or surveillance contexts, where the goal is to detect subclinical variation rather than classify disorder. Missing data are handled according to a uniform rule: for any individual instrument, item-mean imputation is applied for up to 20% of missing items; above this threshold, the instrument score is treated as invalid for that participant and excluded from analyses using that instrument. Participants with more than 30% missing data across the full protocol are flagged for exclusion from primary analyses and examined for systematic patterns that may indicate problems with instrument comprehension or administration. Item-mean imputation and equal-weighted composites are adopted here as operational defaults chosen for field simplicity and cross-site consistency in low-resource implementation contexts, not because they are presented as statistically superior to alternatives. Multiple imputation and full-information maximum likelihood methods are now the recommended standard for handling missing data in psychometric research (Schafer and Graham, 2002) and generally outperform single-value imputation methods such as item-mean substitution, particularly when data are not missing completely at random. Study teams with the statistical infrastructure and software access to implement multiple imputation are encouraged to do so as the primary analytic approach, using the item-mean imputation rule specified here as a documented, replicable fallback for field contexts where this is not feasible, and as a sensitivity comparison in all contexts. Similarly, equal weighting of instruments within domain composites is adopted as a transparent, assumption-light default; it is not intended as a claim that all instruments within a domain contribute equally to the underlying construct, and studies with adequate sample sizes are encouraged to report impact-weighted composites as a sensitivity analysis, as already noted below.

For domain-level composite scores, individual instrument scores within each domain are first standardized using z-scores within the study sample. Domain composites are then computed as the mean of standardized instrument scores within the domain. Equal weighting of instruments within domains is the default; impact-weighted composites using meta-analytic effect size estimates as weights are permitted as sensitivity analyses but should not replace the equal-weighted composite as the primary outcome. The three domain composites, eco-affective, cognitive, and functional, constitute the primary analytic outputs of the full EAHAP and are used in dose-response modeling, early warning detection, and intervention evaluation applications.

### 3.7 Spatial linkage with environmental exposure data

For studies using the EAHAP as the human signal layer of the EAEWS or other spatial exposure-health frameworks, the linkage of EAHAP scores to environmental exposure indices requires careful procedural attention. The primary spatial unit of analysis is the census tract or equivalent administrative unit, which provides geographic specificity sufficient for subnational policy decisions while preserving participant privacy through spatial aggregation. Individual participant data are never linked to environmental data at the individual level; all linkage occurs at the aggregate level after individual spatial assignment is complete and individual identifiers are removed.

Temporal alignment of EAHAP collection with environmental exposure windows is a critical design decision. For studies examining cumulative chronic exposure, the recommended approach is to align the EAHAP collection date with the endpoint of the exposure window, such that a 30-day exposure window captures the 30 days immediately preceding participant assessment. For studies examining acute event impacts, the EAHAP is administered at a fixed interval after the event, with the interval specified in the study protocol. Multiple exposure windows, at 30, 60, and 90 days, can be examined in sensitivity analyses to assess whether effect estimates are stable across exposure window specifications, which constitutes evidence of dose-response robustness.

## 4 Anticipated results

Everything that follows in this section, including specific numerical thresholds for internal consistency, fit indices, inter-instrument correlation patterns, and the shapes of dose-response curves, is an a priori theoretical prediction derived from the EAHAP's conceptual architecture and existing literature on its component constructs. No original data were collected for this manuscript, and none of the values reported below should be read as empirical findings. These predictions are offered as falsifiable, pre-registerable benchmarks against which future multi-site validation studies can be evaluated, and their specificity is intended to support that falsifiability rather than to imply that they have already been observed.

### 4.1 Expected psychometric properties

Each instrument in the EAHAP has established psychometric properties in its original validation context. The primary anticipated result of the EAHAP psychometric evaluation at each new implementation site is replication of acceptable internal consistency (Cronbach's alpha or McDonald's omega above 0.70) for each instrument after cultural adaptation. Factor structures corresponding to published validation studies are expected to replicate in confirmatory factor analyses, with acceptable fit indices (comparative fit index above 0.90, root mean square error of approximation below 0.08). Where instruments are adapted for new contexts, some deviation from published factor solutions is anticipated and should be evaluated through exploratory factor analysis before confirmatory testing.

**Figure 2:**
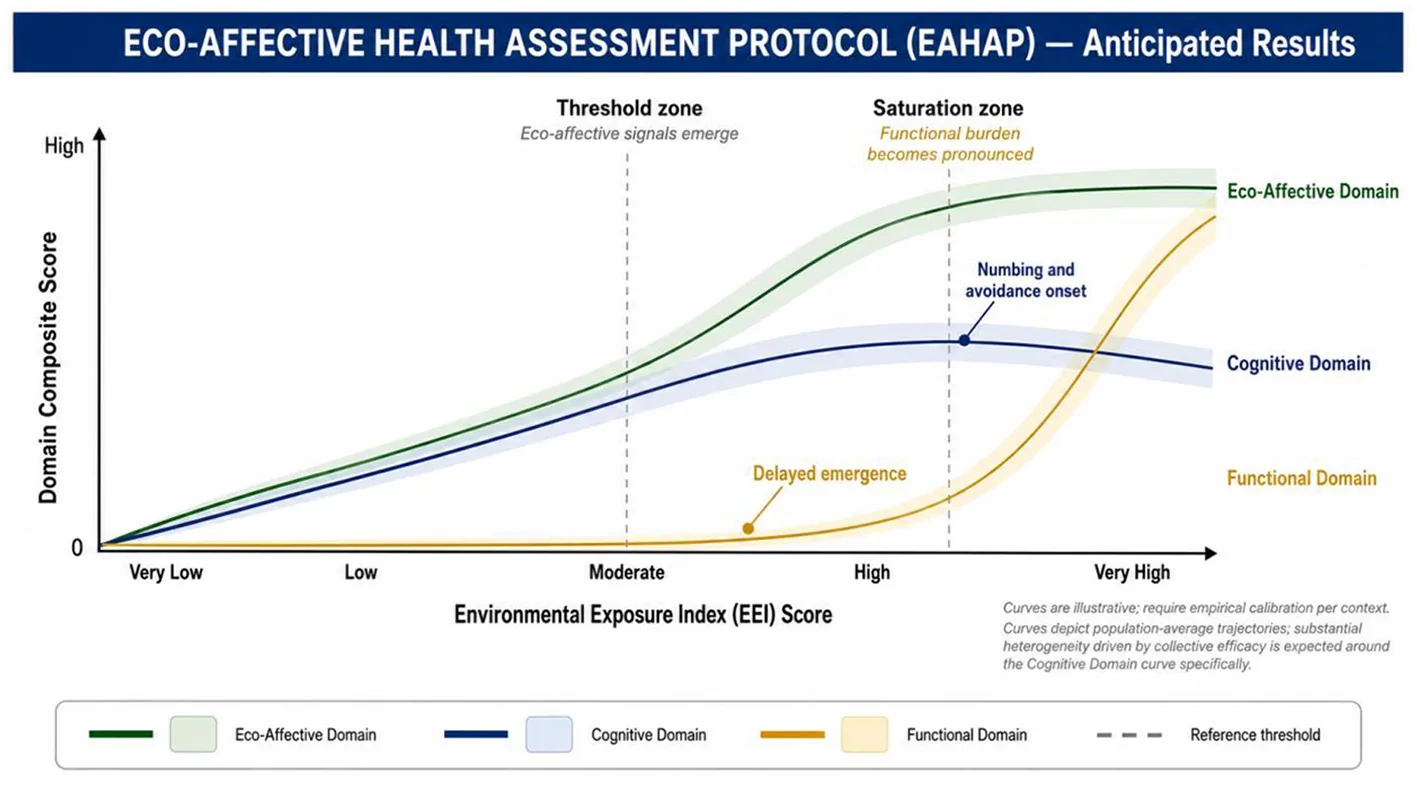
Anticipated dose-response relationships between the Environmental Exposure Index (EEI) and the three EAHAP domain composite scores across the exposure range. The eco-affective domain shows a positive, non-linear relationship with a threshold effect at moderate exposure levels. The cognitive domain shows an initial positive relationship that plateaus or slightly reverses at high exposure levels, reflecting the onset of affective numbing and cognitive avoidance. The functional domain shows a delayed positive relationship that becomes pronounced at exposure levels where eco-affective and cognitive signals are already substantially elevated, consistent with its theoretical positioning as a downstream validation target. Shaded regions indicate a qualitative, illustrative range of plausible variation around each curve and should not be interpreted as an empirically derived confidence interval; no data have been collected to estimate sampling variability, and the width of the shaded regions carries no statistical meaning. Curves are illustrative based on theoretical predictions and available empirical evidence and will require empirical calibration in each implementation context.

At the domain level, moderate positive correlations among instruments within each domain are expected, reflecting genuine construct overlap while confirming that instruments are not redundant. The eco-affective domain is expected to show the strongest inter-instrument correlations, given the theoretical proximity of solastalgia, eco-anxiety, and ecological grief as distinct but related responses to environmental loss and threat. The cognitive domain is expected to show somewhat lower inter-instrument correlations, reflecting the greater conceptual heterogeneity of risk perception, attentional processing, appraisal, and decision-making as constructs. Across domains, moderate positive correlations between the eco-affective and functional domains are expected, with the cognitive domain showing intermediate correlations with both (Figure 2).

### 4.2 Expected dose-response relationships

In studies linking EAHAP scores to environmental exposure indices, a positive dose-response relationship between Environmental Exposure Index scores and eco-affective domain composite scores is the primary expected finding. Based on existing literature on solastalgia, eco-anxiety, and related constructs, the relationship is expected to be non-linear, with a steeper increase in eco-affective burden at higher exposure levels, consistent with a threshold effect above which cumulative environmental stress produces disproportionate affective burden. This non-linearity is one of the theoretically central predictions of the Eco-Affective Health framework and should be examined using flexible modeling approaches such as generalized additive models rather than assumed linear in primary analyses.

The cognitive domain composite is expected to show a more complex relationship with environmental exposure than the eco-affective domain. This added complexity follows directly from the framework's premise, elaborated in section 3.1, that cognitive processing is shaped by prior affective states rather than developing independently of them: across the exposure range, cognitive scores are the net result of two competing processes rather than a single mechanism. At low to moderate exposure levels, risk perception and threat appraisal scores are expected to increase with EEI, reflecting genuine environmental threat awareness. At high exposure levels, particularly in contexts of chronic cumulative exposure, a potential plateau or even decline in risk perception scores may be observed, reflecting the onset of affective numbing and cognitive avoidance as regulatory responses to overwhelming threat. In terms of the pathway shown in Figure 1, this pattern is the empirical signature of the feedback arrow linking affective numbing to risk perception: at low and moderate exposure, that feedback pathway is largely inactive because numbing has not yet accumulated, so risk perception tracks exposure directly; at high, chronic exposure, the feedback pathway becomes active as numbing accumulates, and its attenuating effect on risk perception increasingly offsets, and at sufficiently high exposure may outweigh, the direct threat-detection effect, producing the plateau or reversal. This is conceptually analogous to the affective tipping point dynamics described for related eco-affective systems, in which the same exposure level can produce divergent cognitive and behavioral trajectories depending on whether protective affective resources have already been depleted (Pihkala, 2020; Stanley et al., 2021; Marques, 2026a). This inverted-U or plateau pattern in the cognitive domain at high exposure levels is a theoretically novel prediction of the Eco-Affective Health framework that distinguishes it from linear exposure-response models and has direct implications for early warning system design. These curves represent expected population-average relationships between exposure and each domain; the framework additionally predicts substantial heterogeneity around this average, driven by collective efficacy and social support, such that identical exposure levels may yield divergent cognitive trajectories across subpopulations, with high-collective-efficacy populations expected to show a shallower or absent plateau and low-collective-efficacy populations expected to show an earlier and steeper decline (Marques, 2026a). This moderation should be examined empirically by stratifying or interacting collective efficacy measures with EEI scores in dose-response models, rather than assuming a single population-average curve applies uniformly across contexts. Collective efficacy is not itself included as a core EAHAP instrument in Table 2 or the module selection matrix in Table 4: it is a theorized moderator external to the battery as currently defined, and studies wishing to test this moderation hypothesis must supplement the EAHAP with a validated collective efficacy measure of their own choosing (e.g., a neighborhood or community collective efficacy scale appropriate to the study context), selected and adapted following the same cultural-adaptation procedure specified in section 3.5 for core EAHAP instruments.

### 4.3 Advantages

The principal advantage of the EAHAP over existing approaches is the provision of a theoretically coherent and empirically grounded integrated framework that covers the full range from early affective sensing to functional impact within a single assessment protocol. No existing instrument or protocol covers this range with the combination of theoretical integration, predominantly validated instruments supplemented by one explicitly flagged candidate measure pending validation, and implementable procedures that the EAHAP provides. The modular structure allows researchers with domain-specific questions to use the relevant subset without sacrificing the ability to position their findings within the broader EAHAP framework. The standardized scoring and composite construction procedures enable cross-study synthesis in ways that the current fragmented landscape of eco-affective measurement does not.

A second advantage is the explicit integration of cultural adaptation as a mandatory protocol component rather than an optional supplement. By specifying the adaptation procedure in detail and identifying the constructs most sensitive to cultural variation, the EAHAP enables genuinely cross-cultural research rather than the superficial translation of instruments developed in Western high-income contexts. The compatibility of the EAHAP with spatial exposure data linkage, through its geocoding and temporal alignment procedures, positions it as the only available instrument battery designed from the outset for use in environmental epidemiology and early warning contexts, rather than being adapted for these purposes *post hoc*.

### 4.4 Limitations

The EAHAP in its standard form relies entirely on self-report, which introduces well-documented vulnerabilities to social desirability bias, retrospective distortion, and limitations of introspective access to affective and cognitive states. The ecological validity of self-report instruments is particularly uncertain for attentional processing constructs, where behavioral task performance has been shown to diverge from self-reported attentional tendencies in laboratory studies of environmental threat processing. Researchers requiring high-precision measurement of attentional mechanisms should supplement EAHAP cognitive domain instruments with objective attentional tasks, and the protocol provides guidance on appropriate tasks for laboratory implementations.

The concurrent validity of the EAHAP, specifically the empirical relationships among domains and between domains and environmental exposure, has not yet been established through multi-site longitudinal studies using the full protocol. The anticipated results described above are grounded in the existing literature on individual constructs and the theoretical architecture of the Eco-Affective Health framework, but they constitute predictions rather than established findings. Multi-site validation of the complete EAHAP is the most urgent next step for the field and should be treated as a priority research agenda item by groups adopting the protocol.

Finally, some constructs in the EAHAP, particularly ecological grief and solastalgia, may have limited applicability in populations with very low ecological embeddedness, including urban populations with minimal contact with natural environments and limited place attachment to local ecological features. In these populations, eco-anxiety and adaptive decision-making constructs are likely to show more variance and stronger relationships with environmental exposure than place-based constructs, and module selection should reflect this population characteristic.

## 5 Discussion

The Eco-Affective Health Assessment Protocol addresses a specific and consequential gap in the scientific infrastructure of a rapidly growing field. The proliferation of incompatible instruments measuring the same constructs is not merely an aesthetic problem of scientific tidiness. It is a structural barrier to cumulative knowledge, a source of apparent empirical inconsistency that may reflect measurement differences rather than genuine variation in the phenomena being studied, and a practical obstacle to the translation of eco-affective research into the surveillance systems and policy instruments that the urgency of accelerating environmental change demands. The EAHAP does not solve this problem by introducing yet another set of instruments. It solves it by providing the organizing framework within which existing instruments, the large majority already independently validated and one explicitly flagged as a candidate measure pending validation, can be deployed coherently, compared systematically, and synthesized cumulatively.

The theoretical positioning of the EAHAP within the Eco-Affective Health framework has implications that extend beyond the choice of instruments. By treating affect as a primary regulatory mechanism rather than a downstream outcome, the framework generates specific and testable predictions about the structure of relationships among domains, including the non-linear dose-response patterns described in section 4.2, the potential plateau and reversal of cognitive domain scores at high exposure levels, and the delayed emergence of functional domain effects relative to eco-affective and cognitive signals. These predictions are not merely theoretical elaborations; they are the basis on which the EAHAP can be used to detect early warning signatures of rising environmental mental health burden before functional and clinical thresholds are crossed. The early warning logic of the protocol, formalized in the EAEWS framework, depends precisely on this temporal and causal ordering of the signal layers. This logic is directly analogous to the use of early-warning signals for critical transitions in complex systems more broadly, where measurable changes in system behavior, such as rising variance and slowing recovery from perturbation, precede large-scale regime shifts (Scheffer et al., 2009); the EAHAP applies this same early-warning logic to eco-affective and cognitive signal layers rather than to ecological state variables.

The cognitive domain of the EAHAP deserves particular comment in the context of eco-affective cognition research. The four constructs measured in this domain, environmental risk perception, attentional processing, environmental appraisal, and adaptive decision-making, are not simply add-ons to a primarily affective framework. They are the mechanisms through which ecological belonging or disconnection is actively constructed and sustained in real time. Risk perception determines which environmental signals reach awareness and which are filtered out. Attentional scanning and avoidance determine whether threat information is approached or deflected. Primary appraisal determines how severe the threat is judged to be; secondary appraisal determines whether anything meaningful can be done about it. And adaptive decision-making determines whether the affective and cognitive processing that precedes it results in engagement, avoidance, or fatalistic withdrawal. Without measuring these cognitive mechanisms, eco-affective research can document that people feel distressed about environmental change without being able to explain why some feel distressed and act, while others feel distressed and disengage, and still others appear to feel nothing and neither act nor disengage but simply carry on in a state of functional but affectively impoverished accommodation to degraded conditions.

The modular design of the EAHAP is a practical necessity but also a theoretical commitment. Not all research questions require measurement of all three domains, and the scientific cost of imposing unnecessary measurement burden on participants, in the form of longer protocols that generate higher dropout and lower data quality, outweighs the abstract benefit of comprehensiveness. The module selection matrix in Table 4 is designed to guide researchers toward the minimum viable protocol for their question, while making transparent the tradeoffs involved in domain-specific implementation. Studies that use only the eco-affective domain will be unable to explain cognitive variability in response to comparable exposure. Studies that use only the cognitive domain will be unable to detect the affective early warning signals that often precede measurable cognitive changes. And studies that use only the functional domain will be measuring downstream consequences without access to the upstream processes that generate them. These are not methodological failures but deliberate analytic choices, and they should be acknowledged as such in the discussion of any study using domain-specific rather than full EAHAP implementation.

The cultural adaptation requirements of the EAHAP reflect a genuine epistemological commitment rather than a procedural formality. Eco-affective constructs are not culturally neutral. The experience of solastalgia is shaped by the specific ecological relationships, land-use histories, and place narratives that characterize different cultural contexts, and these vary profoundly across urban and rural environments, Indigenous and non-Indigenous communities, Global North and Global South settings, and societies with different relationships between human identity and ecological belonging. Adapting the EAHAP for use in a new cultural context is not primarily a translation exercise; it is an interpretive process that requires engagement with local environmental psychologists, community members, and researchers who understand how ecological experience is constructed and expressed in that context. The five-step adaptation procedure specified in section 3.5 is the minimum necessary for this interpretive work to produce an instrument that is both structurally comparable to the original and genuinely valid in the new setting.

The most important next step for the field is the empirical validation of the complete EAHAP across multiple sites and cultural contexts. The psychometric properties and inter-domain relationships described in section 4 are predictions grounded in existing literature and theoretical logic, but they require prospective empirical confirmation in studies that administer the full protocol, collect environmental exposure data, and follow participants longitudinally. Such multi-site studies would simultaneously validate the instrument battery, test the theoretical pathway model, and calibrate the dose-response and threshold parameters needed for the EAHAP to function as a genuine early warning system. The development of an international collaborative network of research groups committed to shared EAHAP implementation is, in the view of this paper, the scientific infrastructure investment most likely to accelerate the field of eco-affective health toward the cumulative, cross-contextual, and policy-relevant knowledge base that the scale of the environmental crisis demands.

## Data Availability

The original contributions presented in the study are included in the article/supplementary material, further inquiries can be directed to the corresponding author.
